# Radiologic Axillary Response After Neoadjuvant Chemotherapy in cN2 Breast Cancer: Decision Support for Selective Axillary De-Escalation

**DOI:** 10.3390/cancers18172874

**Published:** 2026-09-05

**Authors:** Mehmet Ali Nazlı, Emel Esmerer, Sümeyye Yeliz Gümüştaş, Ebru Şen, Gökmen Umut Erdem

**Affiliations:** 1 Department of Radiology, Başakşehir Çam and Sakura City Hospital, 34480 Istanbul, Turkey; 2 Department of General Surgery, Başakşehir Çam and Sakura City Hospital, 34480 Istanbul, Turkey; 3 Department of Medical Oncology, Başakşehir Çam and Sakura City Hospital, 34480 Istanbul, Turkey

**Keywords:** breast cancer, cN2 breast cancer, neoadjuvant chemotherapy, axillary ultrasound, targeted axillary dissection, axillary de-escalation, nodal response, pathologic complete response

## Abstract

**Background/Objectives:** Axillary lymph node dissection (ALND) remains common in patients presenting with a high baseline axillary nodal burden despite substantial nodal downstaging after neoadjuvant chemotherapy (NAC). This study evaluated whether post-neoadjuvant radiologic axillary response could support selective axillary de-escalation in patients with clinically staged cN2 breast cancer and pathologically confirmed axillary metastasis. **Methods:** This retrospective single-center cohort included 166 cases treated between October 2020 and August 2024. All had pathologically confirmed axillary metastasis, completed neoadjuvant systemic therapy, underwent post-treatment axillary ultrasound, and received definitive surgery. Radiologic node-negative status (radN0) was defined by restoration of benign nodal morphology. Surgical management and pathological nodal outcomes were evaluated according to radiologic response. **Results:** Radiologic nodal clearance occurred in 61/166 cases (36.7%), while axillary pathological complete response [ypN0(i−)] was achieved in 59/166 (35.5%). ypN0(i−) was observed in 53/61 radN0 cases (86.9%) versus 6/105 radN+ cases (5.7%; *p* < 0.001). Among radN0 cases, 47/61 (77.0%) underwent limited sentinel lymph node biopsy/targeted axillary dissection (SLNB/TAD) without ALND, of whom 43/47 (91.5%) achieved ypN0(i−). Axillary pCR rates varied by molecular subtype, ranging from 20.5% in HR+/HER2− disease to 72.2% in triple-negative disease (*p* < 0.001). No ipsilateral axillary recurrence occurred in the limited SLNB/TAD group during a median follow-up of 36 months. **Conclusions:** Post-neoadjuvant radiologic nodal clearance was strongly associated with pathologically node-negative findings and may support consideration of carefully selected responders with cN2 breast cancer for SLNB/TAD-based staging. Radiologic response should complement, rather than replace, surgical staging and multidisciplinary decision-making.

## Simple Summary

Patients with breast cancer and extensive lymph node involvement in the armpit often undergo axillary lymph node dissection after neoadjuvant treatment, even when the lymph nodes respond well to therapy. This study investigated whether the appearance of axillary lymph nodes on ultrasound after treatment could help identify selected patients with cN2 breast cancer who might be considered for less extensive axillary surgery. Among 166 patients, radiologic normalization of the axillary lymph nodes was strongly associated with the absence of residual nodal disease at surgery. Most patients with favorable radiologic response who underwent limited axillary surgery had no residual tumor in the removed sentinel and targeted lymph nodes. These findings suggest that post-treatment ultrasound may provide useful additional information for multidisciplinary surgical planning. However, imaging should not replace surgical staging and should be interpreted together with tumor biology, treatment response, and intraoperative findings.

## 1. Introduction

Axillary management in breast cancer has progressively shifted from routine axillary lymph node dissection (ALND) toward less extensive surgical strategies aimed at reducing lymphedema, shoulder dysfunction, and other treatment-related morbidity without compromising regional disease control. Sentinel lymph node biopsy (SLNB) has replaced ALND for axillary staging in selected patients with clinically node-negative or limited node-positive disease, and randomized trials have supported omission of completion ALND in carefully selected settings [1,2,3,4,5]. However, these established de-escalation strategies cannot be directly extrapolated to patients presenting with a high axillary nodal burden.

Neoadjuvant chemotherapy (NAC) has further changed axillary management by allowing nodal response to systemic treatment to be evaluated before definitive surgery. Substantial nodal downstaging can occur after NAC, particularly in biologically responsive subtypes such as HER2-positive and triple-negative breast cancer (TNBC) [6]. Prospective studies, including ACOSOG Z1071, SENTINA, and SN FNAC, demonstrated the feasibility of SLNB after NAC in patients with biopsy-proven node-positive disease, although the false-negative rates associated with SLNB alone raised concerns regarding its reliability in this setting [7,8,9].

Retrieval of the previously biopsied and clipped metastatic lymph node in addition to SLNB led to the development of targeted axillary dissection (TAD) [10,11]. By ensuring removal of the initially involved index node, TAD improves the reliability of post-neoadjuvant axillary staging. Several localization techniques have been developed for target-node retrieval, including wire localization, radioactive or magnetic seeds, radar-based markers, intraoperative ultrasound, and tattoo-based approaches. Carbon tattooing is a relatively simple and cost-effective method that facilitates intraoperative identification of the previously involved node without requiring radioactive materials or specialized detection systems [12,13].

Imaging has a central role throughout this pathway. At diagnosis, high-resolution axillary ultrasound enables morphologic assessment, image-guided biopsy, and clip placement in the metastatic index node. After NAC, ultrasound provides dynamic information regarding nodal response, including changes in cortical thickness, nodal shape, and fatty hilar morphology. However, post-treatment ultrasound should not be regarded as a stand-alone replacement for surgical staging. Its more relevant clinical role may be to provide decision support within a multidisciplinary pathway that also considers tumor biology, overall treatment response, clinical findings, and intraoperative assessment [14,15].

Most evidence supporting SLNB or TAD after NAC has been derived from patients with initially limited nodal disease, predominantly cN1 breast cancer. Evidence remains limited for patients presenting with radiologically extensive axillary disease. These patients frequently undergo ALND irrespective of treatment response, although a meaningful proportion may achieve nodal sterilization after contemporary neoadjuvant treatment. A response- and biology-informed assessment could therefore help identify selected patients who might be offered an SLNB/TAD-based approach rather than automatic ALND.

The present study aimed to evaluate the role of post-neoadjuvant radiologic axillary response as a decision-support component for selective axillary de-escalation in patients with cN2 breast cancer. We also assessed the pathologic nodal outcomes of patients offered an SLNB/TAD-based approach, the technical feasibility of target-node localization and retrieval, and the clinical and biologic factors associated with nodal response.

## 2. Materials and Methods

### 2.1. Study Design and Setting

This retrospective cohort study was conducted at a tertiary referral breast cancer center. The study was approved by the institutional ethics committee, and the requirement for written informed consent was waived because of the retrospective design. The study was conducted in accordance with the Declaration of Helsinki and is reported according to the Strengthening the Reporting of Observational Studies in Epidemiology (STROBE) recommendations.

### 2.2. Study Population

Consecutive eligible cases of invasive breast cancer diagnosed between October 2020 and August 2024 were screened. Cases were included when they fulfilled all the following criteria: clinical cN2 disease at initial presentation based on an integrated clinical–radiologic assessment; pathologically confirmed axillary lymph node metastasis by ultrasound-guided biopsy; completion of the planned neoadjuvant systemic treatment; post-treatment radiologic reassessment of the axilla; definitive breast and axillary surgery at the study institution; and availability of complete surgical pathology data.

Cases were excluded when axillary metastasis was not pathologically confirmed, neoadjuvant treatment was incomplete, definitive surgery was not performed, or post-treatment radiologic or surgical pathology data were unavailable. The unit of analysis was the eligible breast–axillary treatment episode. Bilateral or temporally distinct eligible treatment episodes were evaluated as separate clinical cases when each episode fulfilled the inclusion criteria.

### 2.3. Baseline Clinical–Radiologic Nodal Staging and Characterization of Axillary Disease Burden

Baseline clinical nodal stage was assigned according to the AJCC/TNM staging framework [16] using an integrated clinical–radiologic assessment. All patients included in the study were clinically staged as cN2 and had pathologically confirmed axillary lymph node metastasis. Axillary imaging was additionally used to characterize the extent of nodal disease according to the number, distribution, and morphology of suspicious lymph nodes. Within this clinically staged cN2 cohort, extensive radiologic axillary disease burden was characterized by three or more morphologically suspicious axillary lymph nodes and/or confluent or matted-appearing nodal disease involving the level I–II axilla. These imaging features were used to characterize disease burden and were not used as stand-alone criteria for assigning clinical N stage. In particular, multiple suspicious or confluent/matted-appearing nodes on imaging alone were not considered equivalent to the AJCC clinical criterion of fixed or matted lymph nodes.

### 2.4. Baseline Axillary Ultrasound and Nodal Biopsy

At initial diagnosis, all patients underwent high-resolution axillary ultrasound. Baseline and post-treatment axillary imaging assessments were performed by three dedicated breast radiologists, each with at least 10 years of experience in breast imaging. The final radiologic nodal classification was determined by consensus among the three radiologists. Examinations were performed using a Philips EPIQ Elite ultrasound system equipped with an eL18-4 high-frequency linear-array transducer (4–18 MHz; Philips Healthcare, Best, The Netherlands).

The entire axilla was assessed, including the number, distribution, and morphology of suspicious lymph nodes. The most representative and technically accessible suspicious node was sampled using ultrasound-guided core needle biopsy or fine-needle aspiration. Metastatic involvement was pathologically confirmed in at least one morphologically suspicious axillary lymph node in all included cases. The pathologically confirmed metastatic node was designated the index node and was marked before initiation of NAC using a Geotek^®^ gold seed metallic marker (Geotek Medical, Ankara, Türkiye) Additional morphologically suspicious lymph nodes contributing to the imaging-defined axillary disease burden were assessed radiologically and were not routinely biopsied individually. Whenever technically feasible, activated charcoal tattooing was performed under ultrasound guidance during the same session as clip placement.

### 2.5. Molecular Subtype Classification

Tumors were classified into four clinically relevant molecular subtypes according to hormone receptor and HER2 status: HR+/HER2−, HR+/HER2+, HR−/HER2+, and TNBC. Hormone receptor positivity was defined as estrogen receptor or progesterone receptor expression in at least 1% of tumor cells. HER2 status was determined by immunohistochemistry and, when indicated, in situ hybridization according to the contemporaneous ASCO/CAP recommendations.

### 2.6. Neoadjuvant Treatment and Timing of Surgery

All included patients completed the planned neoadjuvant systemic treatment before definitive surgery. Treatment regimens were selected according to molecular subtype and the institutional multidisciplinary breast cancer protocol. All patients with HER2-positive disease received standard dual HER2-targeted therapy.

Pembrolizumab was not routinely administered to patients with TNBC during the study period. The study was not designed to evaluate the efficacy of individual systemic treatment regimens; rather, it assessed the role of post-treatment radiologic response and tumor biology in supporting selective axillary surgical planning. Definitive breast and axillary surgery was performed 4–8 weeks after completion of neoadjuvant treatment, most commonly within 4–5 weeks.

### 2.7. Post-Neoadjuvant Radiologic Axillary Assessment

After completion of neoadjuvant treatment and before definitive surgery, the axilla was reassessed using high-resolution ultrasound. Post-treatment evaluation included the clipped index node and the remainder of the ipsilateral axilla. Nodal morphology was evaluated according to cortical thickness, hilar preservation, nodal shape, and residual suspicious features. Short-axis measurements were recorded when applicable; however, the final case-level radiologic classification incorporated both nodal size and morphology rather than RECIST measurements alone.

Radiologic node-negative status (radN0) was defined as restoration of benign nodal morphology, including cortical thickness of 3 mm or less, preservation or restoration of the fatty hilum, and absence of persisting morphologically suspicious axillary nodes. Radiologic node-positive status (radN+) was defined by the presence of at least one lymph node with persistent cortical thickening greater than 3 mm, loss or replacement of the fatty hilum, round morphology, asymmetric cortical bulging, or another feature considered suspicious for residual nodal disease. The final post-treatment radiologic nodal status was assigned by consensus among the three breast radiologists and was used as a multidisciplinary decision-support component rather than as a stand-alone diagnostic test.

### 2.8. Multidisciplinary Surgical Decision-Making

**Figure 1:**
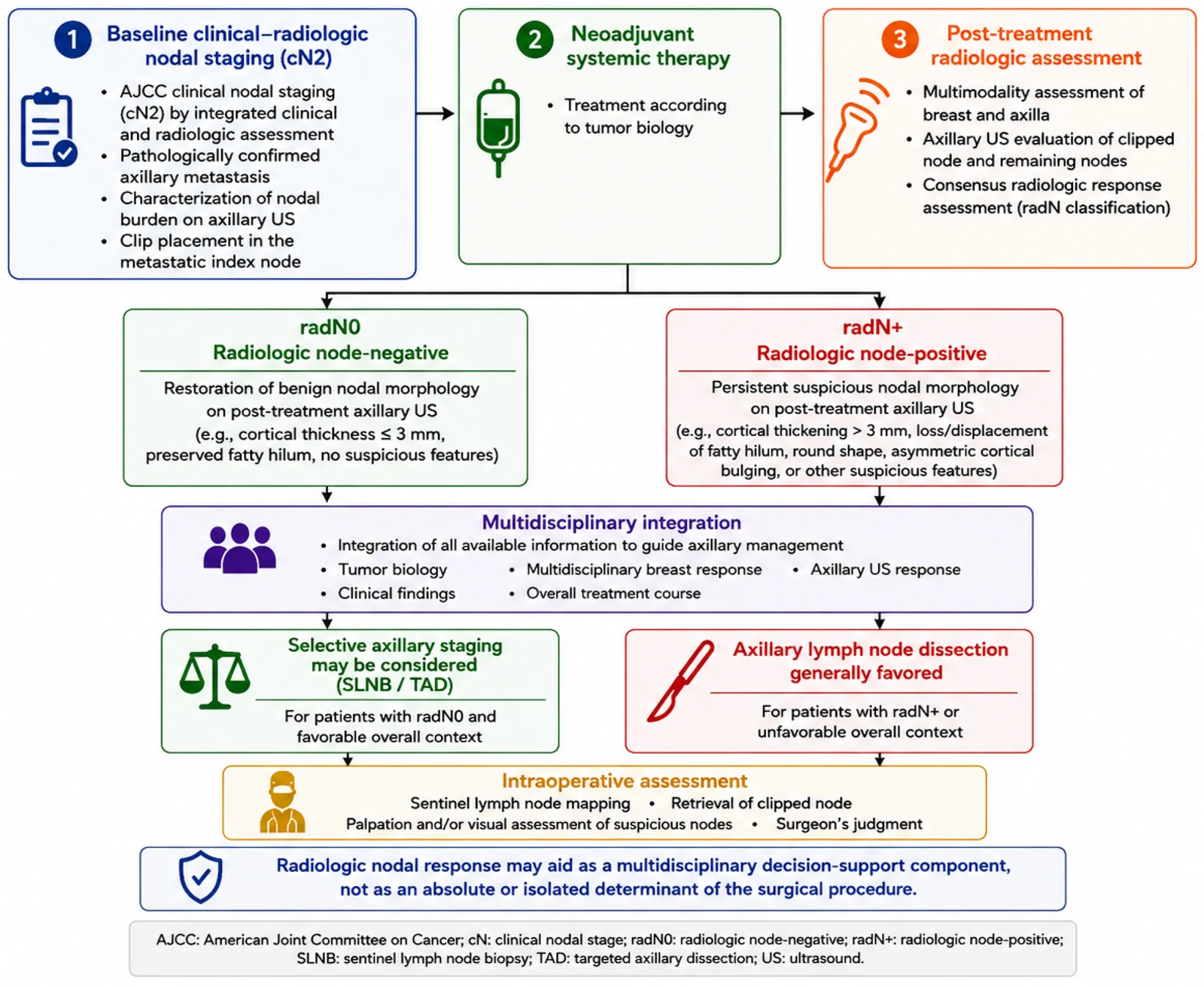
Multidisciplinary decision pathway for response-adapted axillary management after neoadjuvant therapy in cN2 breast cancer.

Following post-treatment assessment, axillary management was discussed at the multidisciplinary breast cancer meeting. Radiologic nodal response was interpreted together with molecular subtype, primary tumor response, clinical findings, and the overall treatment course. Restoration of benign nodal morphology provided additional confidence for considering selective axillary surgery in patients who would otherwise commonly undergo ALND because of their initial high nodal burden. However, the multidisciplinary recommendation did not exclusively determine the definitive extent of surgery. The final axillary procedure was also influenced by sentinel lymph node mapping, intraoperative palpation, identification and retrieval of the marked target node, and the operating surgeon’s assessment. The multidisciplinary and intraoperative decision pathway is illustrated in Figure 1.

### 2.9. Target-Node Localization

The pathologically confirmed metastatic index node was marked with a clip before initiation of NAC. Whenever technically feasible, activated charcoal tattooing was performed under ultrasound guidance during the same session as clip placement, with 0.5–1.0 mL of activated charcoal suspension injected into or immediately adjacent to the nodal cortex.

Before surgery, the axilla was systematically reassessed by ultrasound. The location of the clipped/marked target node within the axilla and its depth from the skin were documented, and its exact projection onto the overlying skin was topographically marked to facilitate surgical identification.

When additional localization was required and the target node could be reliably identified sonographically, ultrasound-guided carbon tattooing was performed. If the target node could not be reliably identified on ultrasound, CT-guided localization and carbon tattooing were used as a rescue technique. The marked target node was subsequently removed during surgery, and retrieval of the previously clipped index node was confirmed by specimen radiography.

### 2.10. Targeted Axillary Dissection

TAD was defined as the combined removal of the previously pathologically confirmed and marked metastatic index node together with SLNB. Sentinel lymph node mapping was performed according to institutional surgical practice using a single- or dual-tracer technique. The study was not designed to compare the performance of individual mapping or localization techniques.

### 2.11. Extent of Axillary Surgery

In patients undergoing SLNB/TAD after NAC, institutional practice was to aim for retrieval of at least three axillary lymph nodes whenever technically feasible, including the previously pathologically confirmed and marked target node.

Sentinel lymph nodes and the targeted clipped node were routinely evaluated by intraoperative frozen-section examination in patients undergoing axillary surgery after NAC, in accordance with institutional practice. Frozen-section findings were considered together with sentinel lymph node mapping, confirmation of target-node retrieval, palpation findings, and other intraoperative findings when determining whether completion ALND was indicated.

For analysis, the final axillary procedures were categorized as limited axillary surgery without ALND, limited surgery followed by completion ALND, or primary ALND. Limited axillary surgery consisted of SLNB with targeted retrieval of the marked index node without subsequent ALND. Completion ALND followed initial SLNB/target-node retrieval because of intraoperative, mapping, or pathological considerations. Primary ALND was a planned dissection performed without an initial de-escalated surgical approach.

A planned limited procedure could be extended to ALND in the presence of unsuccessful sentinel mapping, inability to confirm retrieval of the clipped target node, palpable or visually suspicious lymph nodes, or other intraoperative findings considered concerning for residual nodal disease. Primary ALND was generally performed in patients with persistent radiologic nodal abnormalities, clinically extensive residual nodal disease, or other multidisciplinary or surgical considerations indicating a high probability of residual axillary involvement. Radiologic nodal status was not used as an absolute or isolated determinant of the surgical procedure.

### 2.12. Pathological Evaluation and RCB

All sentinel, targeted, and non-sentinel axillary lymph nodes were evaluated by dedicated breast pathologists according to institutional protocols. The total number of excised nodes, number of metastatic nodes, and final post-neoadjuvant nodal stage were recorded.

Axillary pathologic complete response was defined as the absence of residual invasive carcinoma, micrometastases, or isolated tumor cells in all surgically retrieved axillary nodes, including the previously pathologically confirmed target node. Cases with isolated tumor cells were classified separately as ypN0(i+) and were not included in the axillary pathologic complete response group. Residual nodal disease was categorized according to the post-neoadjuvant ypN classification.

Residual cancer burden (RCB) was assessed on the definitive surgical specimen according to established RCB methodology, incorporating the dimensions of the residual primary tumor bed, overall tumor cellularity, proportion of in situ disease, number of involved axillary lymph nodes, and size of the largest nodal metastasis. RCB was categorized as RCB-0, RCB-I, RCB-II, or RCB-III.

### 2.13. Study Outcomes

The primary outcome was the proportion of radN0 cases that underwent limited SLNB/TAD-based axillary surgery without completion ALND. The pathological findings of this selectively managed group were assessed to describe the feasibility and clinical yield of the response-adapted pathway.

Secondary outcomes included the overall axillary ypN0 rate; nodal sterilization according to radiologic nodal response and molecular subtype; frequency of completion or primary ALND; frequency of ypN0 among patients undergoing ALND; feasibility of ultrasound- or CT-guided clipped-node localization; successful retrieval of the marked index node; factors associated with nodal sterilization; and ipsilateral axillary recurrence during the available follow-up. The study was not designed as a formal diagnostic-accuracy validation of post-neoadjuvant ultrasound or to establish the definitive oncologic safety of omitting ALND.

### 2.14. Follow-Up

Clinical and imaging follow-up data were obtained from institutional records. Follow-up duration was calculated from definitive surgery to the most recent clinical assessment or documented recurrence. Ipsilateral and contralateral axillary recurrences were recorded separately. Because of the retrospective design and limited number of regional events, recurrence findings were interpreted descriptively and were not considered definitive evidence of comparative oncologic safety.

### 2.15. Statistical Analysis

Continuous variables were assessed for distribution and summarized as mean ± standard deviation or median with interquartile range, as appropriate. Categorical variables were reported as frequencies and percentages. Clinical, radiologic, biological, and pathological characteristics were compared between radN0 and radN+ groups and between ypN0 and ypN+ groups. Categorical variables were compared using the chi-square test or Fisher’s exact test. Continuous variables were compared using the independent-samples *t*-test or Mann–Whitney U test, as appropriate.

The association between post-treatment radiologic nodal status and pathologic nodal response was assessed descriptively and using contingency analyses. For binary radiologic–pathologic concordance analyses, ypN0 and ypN0(i+) were categorized as pathologically node-negative, whereas ypN1–3 were categorized as pathologically node-positive. Because the extent of surgical verification varied according to multidisciplinary and intraoperative selection, sensitivity, specificity, predictive values, and false-negative rate were not considered primary performance measures. All tests were two-sided, and *p* < 0.05 was considered statistically significant. Statistical analyses were performed using IBM SPSS Statistics, version 27.0 (IBM Corp., Armonk, NY, USA), and R, version 4.3.2 (R Foundation for Statistical Computing, Vienna, Austria).

## 3. Results

### 3.1. Study Cohort and Baseline Characteristics

A total of 166 eligible breast–axillary treatment cases with clinically staged cN2 disease and pathologically confirmed axillary metastasis were included in the final analysis. All cases completed the planned neoadjuvant systemic treatment and subsequently underwent definitive breast and axillary surgery. The mean age was 49.0 ± 11.7 years (range, 25–77 years).

Invasive carcinoma of no special type (NST) was the predominant histologic subtype and was present in 149 cases (89.8%). Invasive lobular carcinoma was diagnosed in 10 cases (6.0%), and rare histologic variants were present in seven (4.2%). According to hormone receptor and HER2 status, 88 tumors (53.0%) were classified as HR+/HER2−, 37 (22.3%) as HR+/HER2+, 23 (13.9%) as HR−/HER2+, and 18 (10.8%) as TNBC. All patients with HER2-positive disease received standard dual HER2-targeted therapy. Pembrolizumab was not routinely administered in the TNBC subgroup during the study period. Definitive surgery was performed 4–8 weeks after completion of neoadjuvant treatment, most commonly within 4–5 weeks. Clinicopathologic and treatment-response characteristics according to post-treatment radiologic nodal status are summarized in Table 1.

### 3.2. Primary Tumor Response

Following neoadjuvant treatment, complete radiologic response of the primary breast tumor was observed in 36 cases (21.7%), partial response in 112 (67.5%), stable disease in 15 (9.0%), and progressive disease in three (1.8%). The overall objective response rate, defined as complete or partial response, was 89.2%.

Residual cancer burden was classified as RCB-0 in 35 cases (21.1%), RCB-I in 16 (9.6%), RCB-II in 60 (36.1%), and RCB-III in 55 (33.1%). Overall, 51 cases (30.7%) had RCB-0/I disease, whereas 115 (69.3%) had RCB-II/III disease.

### 3.3. Post-Treatment Radiologic Axillary Response

After completion of neoadjuvant treatment, radiologic nodal clearance (radN0) was observed in 61 of 166 cases (36.7%). Persistent morphologically suspicious axillary nodes (radN+) were present in 105 cases (63.3%). Age did not differ significantly between the radN0 and radN+ groups (48.4 ± 12.6 vs. 49.3 ± 11.2 years, respectively; *p* = 0.626). In contrast, molecular subtype distribution differed significantly according to post-treatment radiologic nodal status (*p* < 0.001).

Radiologic nodal clearance occurred in 23 of 88 HR+/HER2− cases (26.1%), 12 of 37 HR+/HER2+ cases (32.4%), 13 of 23 HR−/HER2+ cases (56.5%), and 13 of 18 TNBC cases (72.2%). Thus, restoration of radiologically benign nodal morphology was more frequent in HR−/HER2+ and triple-negative tumors than in hormone receptor-positive tumors.

### 3.4. Breast and Axillary Surgical Management

Breast-conserving surgery was performed in 103 cases (62.0%), whereas 63 (38.0%) underwent mastectomy. The final axillary surgical approach consisted of limited SLNB/TAD-based surgery without ALND in 53 cases (31.9%), limited axillary surgery followed by completion ALND in 14 (8.4%), and primary ALND in 99 (59.6%).

Across the entire cohort, the mean number of excised axillary lymph nodes was 12.5 ± 7.1, with a median of 12.5 and a range of 1–28. In cases managed with limited SLNB/TAD-based surgery without ALND, the mean number of excised nodes was 4.6 ± 1.9, with a median of 4 and a range of 1–9. The mean number of excised nodes was 12.1 ± 4.0 in the completion ALND group and 16.9 ± 5.3 in the primary ALND group.

### 3.5. Radiologic Response and Selection for Axillary De-Escalation

Among the 61 radN0 cases, 47 (77.0%) underwent limited SLNB/TAD-based axillary surgery without ALND. Six radN0 cases (9.8%) underwent limited surgery followed by completion ALND, and eight (13.1%) underwent primary ALND because of multidisciplinary, clinical, or intraoperative considerations.

**Figure 2:**
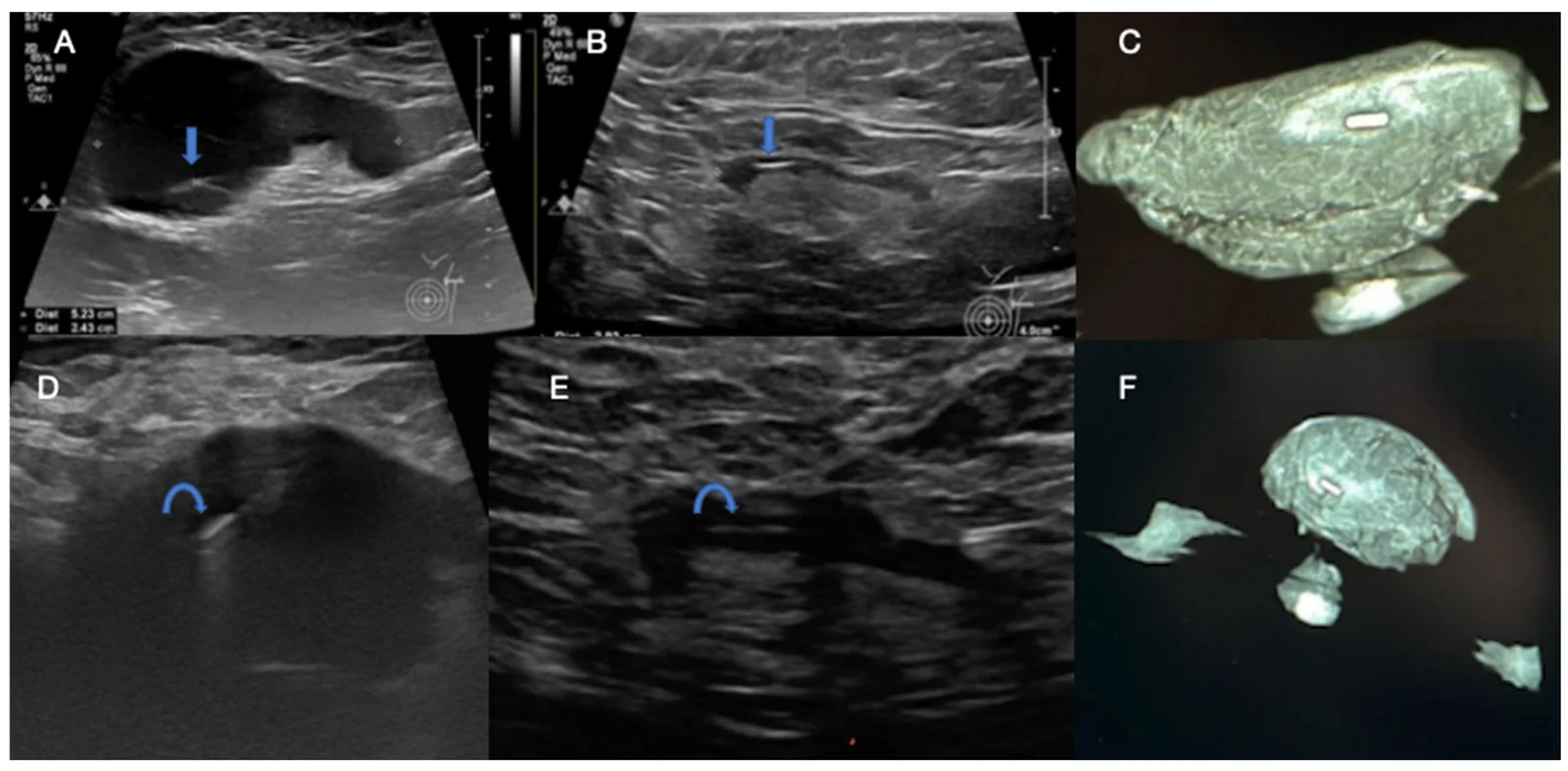
Representative radiologic–pathologic concordance after neoadjuvant therapy. (**A**) Pre-treatment ultrasound of the radN0/ypN0 case shows the biopsy-proven metastatic axillary lymph node with a clip in the nodal cortex (straight arrow). (**B**) Post-treatment ultrasound of the same case demonstrates restoration of benign nodal morphology, with cortical thinning and restoration of the fatty hilum; the clip remains visible within the cortex (straight arrow), consistent with radN0. (**C**) Specimen mammography confirms successful retrieval of the clipped lymph node; final pathology demonstrated ypN0. (**D**) Pre-treatment ultrasound of the radN+/ypN+ case shows the biopsy-proven metastatic axillary lymph node with a clip placed in the cortex (curved arrow). (**E**) Post-treatment ultrasound of the same case demonstrates persistent asymmetric cortical thickening, with the clip visible within the cortex (curved arrow), consistent with radN+. (**F**) Specimen mammography confirms successful retrieval of the clipped lymph node; final pathology demonstrated residual nodal disease (ypN+).

Among the 105 radN+ cases, six (5.7%) underwent limited axillary surgery without ALND, eight (7.6%) underwent limited surgery followed by completion ALND, and 91 (86.7%) underwent primary ALND. These findings demonstrate that post-treatment radiologic nodal status was not used as an absolute determinant of the final surgical procedure. Radiologic nodal clearance provided support for considering an SLNB/TAD-based approach, whereas the final extent of surgery remained influenced by multidisciplinary assessment, sentinel mapping, target-node retrieval, palpation findings, and the operating surgeon’s intraoperative judgment. Representative imaging and surgical–pathological pathways of a patient achieving radiologic nodal clearance (radN0) and a patient with persistent radiologically suspicious nodal disease (radN+) are illustrated in Figure 2. The distribution of final axillary procedures and pathological nodal outcomes according to post-treatment radiologic nodal status is presented in Table 2.

### 3.6. Pathologic Nodal Response

Axillary pathologic complete response was achieved in 59 of 166 cases (35.5%). Four additional cases (2.4%) had isolated tumor cells and were classified separately as ypN0(i+). Residual nodal disease was categorized as ypN1 in 61 cases (36.7%), ypN2 in 27 (16.3%), and ypN3 in 15 (9.0%). High residual nodal burden, defined as ypN2–3, was therefore present in 42 cases (25.3%).

Radiologic nodal clearance was strongly associated with nodal sterilization. ypN0 was observed in 53 of 61 radN0 cases (86.9%), compared with six of 105 radN+ cases (5.7%; *p* < 0.001). In the binary radiologic–pathologic comparison, five radN0 cases had residual nodal metastasis, whereas seven radN+ cases were pathologically node-negative. The clinicopathologic characteristics of these discordant cases are provided in Table S1.

### 3.7. Pathologic Outcomes of Selective Axillary Surgery

Among the 47 radN0 cases managed with limited SLNB/TAD-based surgery without ALND, 43 (91.5%) achieved ypN0. Of the remaining four cases, three were classified as ypN0(i+) and one as ypN1. Thus, most patients selected for limited axillary surgery had no residual nodal disease identified in the retrieved sentinel and targeted nodes.

In the overall limited SLNB/TAD cohort, 52 of 53 patients (98.1%) had three or more axillary lymph nodes excised. Only one patient (1.9%) had fewer than three nodes retrieved, with a single node excised. The median number of excised nodes was 4 (range, 1–9).

Among the six radN0 cases undergoing limited surgery followed by completion ALND, two had ypN0 and four had residual nodal disease. All eight radN0 cases undergoing primary ALND had ypN0. Among the 91 radN+ cases undergoing primary ALND, six (6.6%) had ypN0, one had ypN0(i+), and 84 (92.3%) had residual ypN1–3 disease. Pathological nodal outcomes and the number of excised nodes according to the final axillary procedure are summarized in Table 3.

**Figure 3:**
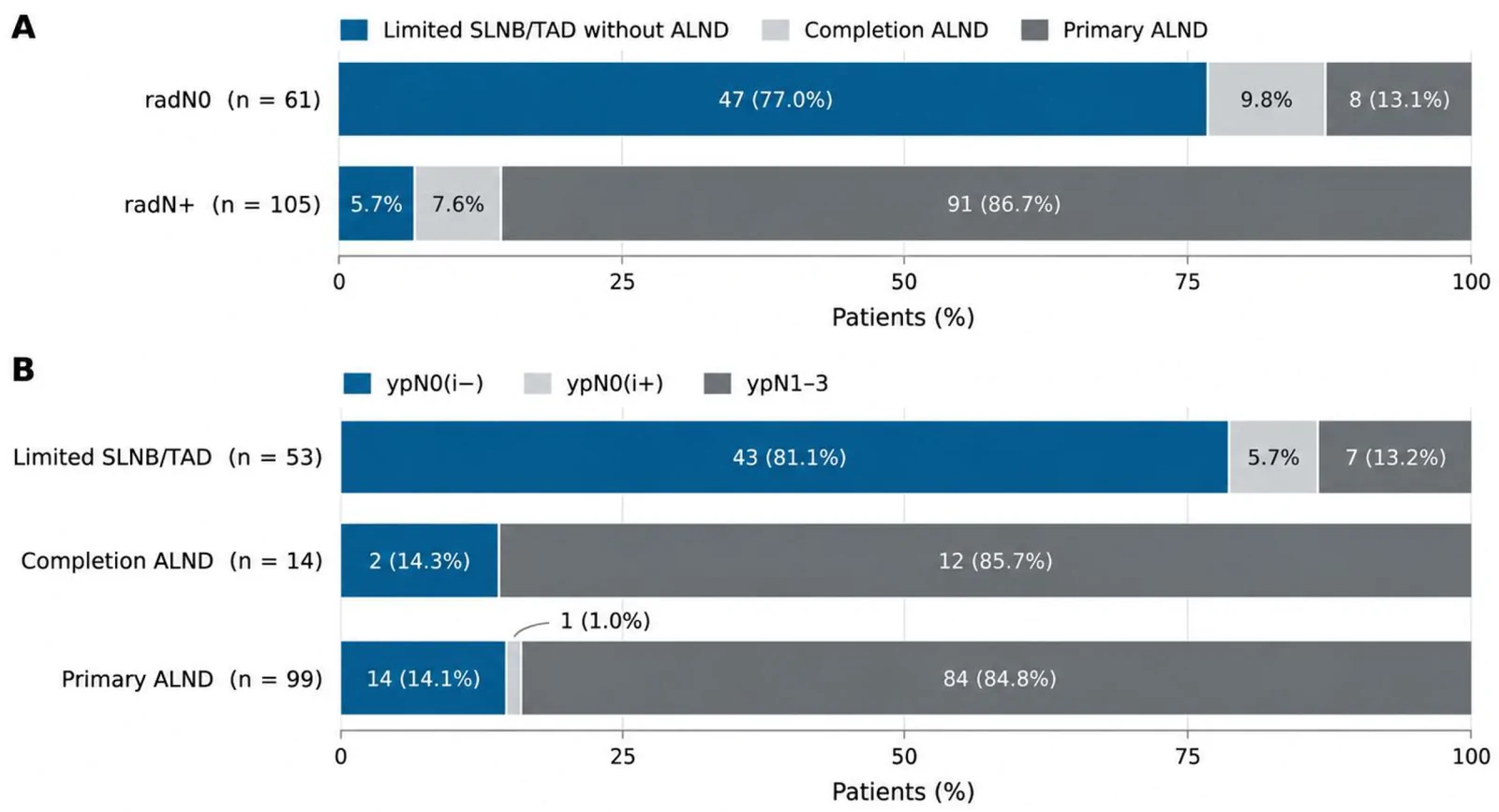
Axillary surgery and pathological nodal outcome. (**A**) Final axillary procedure according to post-treatment radiologic nodal status. (**B**) Pathological nodal outcome according to the final axillary procedure. Values are presented as *n* (%) within each group; narrow segments show percentages only. radN0, radiologically node-negative; radN+, radiologically node-positive; ypN0(i−), no residual nodal disease; ypN0(i+), isolated tumor cells; ypN1−3, residual nodal metastasis; ALND, axillary lymph node dissection; SLNB, sentinel lymph node biopsy; TAD, targeted axillary dissection.

When evaluated according to the final axillary procedure, ypN0 was present in 43 of 53 cases (81.1%) managed with limited SLNB/TAD-based surgery without ALND, two of 14 (14.3%) undergoing completion ALND, and 14 of 99 (14.1%) undergoing primary ALND. These differences reflect preoperative multidisciplinary and intraoperative selection and should not be interpreted as a direct comparison of the oncologic effectiveness of the surgical procedures. The relationship among post-treatment radiologic nodal status, final axillary surgical procedure, and pathological nodal outcome is illustrated in Figure 3.

### 3.8. Molecular Subtype and Nodal Sterilization

Pathologic nodal response differed significantly among molecular subtypes (*p* < 0.001). ypN0 was achieved in 18 of 88 HR+/HER2− tumors (20.5%), 15 of 37 HR+/HER2+ tumors (40.5%), 13 of 23 HR−/HER2+ tumors (56.5%), and 13 of 18 triple-negative tumors (72.2%).

The highest nodal sterilization rate was observed in TNBC, followed by HR−/HER2+ disease, whereas HR+/HER2− tumors demonstrated the lowest ypN0 rate. These findings support interpreting post-treatment radiologic nodal response together with tumor biology when considering selective axillary surgery. Radiologic nodal clearance and ypN0 rates according to molecular subtype are summarized in Table 4.

### 3.9. Target-Node Localization Feasibility

Among the 61 radN0 cases, the target node was successfully identified sonographically in 55 (90.2%). In the remaining six cases (9.8%), reliable ultrasound identification was not possible and CT-guided carbon tattooing was used as a rescue localization technique. The marked target node was subsequently retrieved during targeted axillary surgery. These findings are reported as procedural feasibility observations and not as a comparison of localization technologies.

### 3.10. Regional Recurrence During Follow-Up

The median available follow-up was 36 months, with a range of 5–60 months. Ipsilateral axillary recurrence occurred in two cases (1.2%), and contralateral axillary recurrence occurred in two (1.2%). All four axillary recurrence events occurred in patients who had undergone primary ALND and had persistent high-burden nodal disease classified as ypN2 or ypN3. No recorded ipsilateral axillary recurrence occurred in the limited SLNB/TAD-based surgery group during the available follow-up.

Because of the limited number of regional events, retrospective follow-up, and differences in baseline risk and surgical selection, these findings were interpreted descriptively and were not considered definitive evidence of comparative oncologic safety.

## 4. Discussion

One of the major goals of NAC is to reduce axillary disease burden and thereby allow less extensive, patient-centered surgery in appropriately selected patients. ACOSOG Z1071, SENTINA, and SN FNAC established the feasibility of sentinel lymph node biopsy after neoadjuvant therapy in patients presenting with node-positive disease, while removal of the clipped node and the subsequent development of targeted axillary dissection improved the reliability of post-treatment axillary staging [7,8,9,10,11]. In patients who initially present with cN1 disease and demonstrate nodal downstaging, SLNB- and TAD-based strategies have consequently become an increasingly accepted component of multidisciplinary practice at dedicated breast centers [17,18]. Evidence remains less established for patients presenting with cN2 disease, who are still commonly directed to ALND irrespective of their response to systemic therapy.

In the present cohort, a clinically relevant proportion of patients with clinically staged cN2 disease and pathologically confirmed axillary metastasis achieved substantial nodal downstaging after neoadjuvant treatment. Axillary ypN0 was documented in 35.5% of the overall cohort. Radiologic nodal clearance occurred in 36.7% and was closely associated with nodal sterilization: 86.9% of radN0 cases were ypN0, compared with only 5.7% of radN+ cases. Of the 61 patients classified as radN0, 47 (77.0%) underwent limited SLNB/TAD-based surgery without ALND. No residual disease was found in the retrieved sentinel and targeted nodes in 43 of these 47 patients (91.5%). These results indicate that a response-adapted multidisciplinary assessment may allow selected patients, who might otherwise undergo ALND solely because of initial high-burden node-positive status, to be considered for a less invasive axillary procedure. Importantly, the high proportion of ypN0 findings among patients selected for limited SLNB/TAD reflects enrichment through multidisciplinary and intraoperative selection. It should therefore not be interpreted as evidence of superiority of limited axillary surgery over ALND or as proof of the oncologic safety of ALND omission.

Limited axillary surgery in this study did not consist of SLNB alone. TAD combined sentinel node biopsy with targeted retrieval of the index node that had been pathologically confirmed as metastatic and clipped before treatment. Removal of the initially involved node allows direct pathological assessment of a site known to have contained metastasis and addresses a key limitation of SLNB alone in patients who were node-positive at presentation [7,8,9,10,11]. Post-treatment radN0 status was not, however, used as an automatic indication to omit ALND. Radiologic response was considered alongside molecular subtype, primary tumor response, clinical findings, sentinel mapping, successful retrieval of the target node, intraoperative palpation, the presence of visually suspicious nodes, and the surgeon’s operative assessment. In this setting, radN0 served as decision-support information for offering limited surgical staging rather than as an independent criterion for eliminating axillary surgery.

The distribution of axillary procedures illustrates how this strategy operated in routine practice. Although 47 radN0 patients underwent SLNB/TAD without ALND, six underwent completion ALND after limited surgery and eight proceeded directly to primary ALND. Decisions to extend surgery were influenced by multidisciplinary review and operative findings, including inadequate sentinel mapping or failure of tracer uptake, uncertainty regarding target-node retrieval, palpable or visually suspicious nodes, and the surgeon’s intraoperative judgment. The final procedure therefore reflected the combined interpretation of preoperative and intraoperative information rather than radiologic status alone.

Notably, all eight radN0 patients who underwent primary ALND were ultimately found to have ypN0 disease. This observation may reflect understandable reluctance to depart from conventional ALND in patients who initially presented with cN2 breast cancer, particularly when response-adapted management has not yet been firmly established for this group. It also suggests that some patients with favorable radiologic and biological responses may receive more extensive axillary surgery than their final pathological findings would appear to require. These procedures cannot be considered inappropriate in retrospect, as treatment allocation was not randomized and the operative decision incorporated factors beyond imaging. The relevant implication is not that ALND should routinely be omitted in every radN0 patient, but that patients with a favorable response should not be excluded automatically from consideration for SLNB/TAD solely because they presented with cN2 breast cancer.

Conversely, four of the 47 radN0 patients managed without ALND did not achieve ypN0: three had isolated tumor cells [ypN0(i+)] and one had residual ypN1 disease. This discordance is clinically important. Restoration of benign nodal morphology after treatment does not necessarily indicate complete pathological sterilization. Post-treatment ultrasound cannot replace surgical staging, and radN0 should not be used in isolation to justify omission of all axillary surgery. Persistent abnormal nodal morphology provided complementary information: only 5.7% of radN+ patients had ypN0, and 84 of the 91 radN+ patients who underwent primary ALND had residual ypN1–3 disease. Thus, radN0 may help identify patients in whom accurate limited staging can be attempted, whereas radN+ remains a relevant warning sign for residual disease during multidisciplinary planning. Because the extent of pathological verification differed among the surgical groups, these findings should not be interpreted as formal estimates of ultrasound sensitivity, specificity, or false-negative rate. The strong association between radN0 and pathologically node-negative findings should not be interpreted as evidence that post-treatment axillary ultrasound independently predicts complete nodal response. Molecular subtype and primary breast tumor response were also strongly associated with treatment response and may partly account for the observed radiologic-pathologic relationship. Accordingly, post-treatment axillary ultrasound should be interpreted as one component of an integrated multidisciplinary assessment rather than as a stand-alone predictor of pathological nodal status. Recent prospective AXSANA data similarly demonstrated limitations in the diagnostic performance of post-neoadjuvant axillary ultrasound when used alone and support interpretation of axillary imaging together with tumor biology and breast response [19].

Recent oncologic outcome data provide a broader context for response-adapted axillary management. In the multicenter cohort reported by Montagna et al., which included 1144 patients treated with SLNB or TAD without ALND after nodal downstaging, the five-year rates of axillary, locoregional, and any invasive recurrence were 1.0%, 2.7%, and 10%, respectively [20]. The marked difference between axillary and overall invasive recurrence suggests that subsequent disease events are more often driven by tumor biology and systemic relapse than by isolated failure in the treated axilla. Similarly, the prospective SenTa registry found no significant difference in recurrence or mortality between patients undergoing TAD alone and those undergoing TAD followed by ALND [21]. Most participants in these studies, however, had cN1 disease or a limited number of suspicious nodes, which restricts direct extrapolation to cN2 disease.

The recent MARI protocol data are particularly relevant because they included patients with a higher initial nodal burden, defined by at least four involved axillary nodes on baseline FDG-PET/CT. Among selected patients with a responsive marked MARI node who omitted ALND and received locoregional radiotherapy, the axillary recurrence rate was 2.9%, while five-year invasive disease-free survival and overall survival were 89% and 95%, respectively [22]. Other contemporary studies combining imaging-tailored axillary surgery with adjuvant treatment have likewise reported low regional recurrence rates, with subsequent events occurring more often as distant rather than isolated nodal relapse [22,23]. Collectively, these data suggest that a high initial nodal burden should not invariably rule out consideration of a response-adapted approach. They do not, however, establish omission of ALND as a routine standard for cN2 disease. Recent data have also extended the evaluation of targeted axillary dissection to patients with a higher baseline nodal burden, supporting further investigation of response-adapted axillary staging beyond conventional low-burden node-positive populations [24].

The contribution of radiotherapy is central to the interpretation of these findings. Contemporary axillary de-escalation does not simply replace ALND with an absence of regional treatment. In many published cohorts, systemic therapy, limited surgical staging, and appropriately selected regional radiotherapy act as complementary components of disease control [22,23,24,25,26,27]. By contrast, NSABP B-51/RTOG 1304 showed that additional regional nodal irradiation did not improve recurrence or survival outcomes in patients who initially had cN1 disease and achieved ypN0 after neoadjuvant chemotherapy [28]. This result reinforces the prognostic relevance of treatment response and supports tailoring both surgery and radiotherapy rather than automatically escalating regional treatment. As the B-51 population was limited to cN1 disease, the findings should not be applied directly to patients presenting with cN2 disease.

The follow-up findings in our cohort are consistent with this evolving evidence but cannot independently establish oncologic safety. No ipsilateral axillary recurrence was recorded in the limited SLNB/TAD group during a median follow-up of 36 months. The observed axillary events occurred among patients who had undergone primary ALND and had persistent ypN2–3 disease. This pattern suggests that regional and systemic recurrence may be influenced more strongly by residual disease burden and tumor biology than by the number of axillary nodes removed. Nevertheless, the small number of events, limited follow-up, differences in treatment selection, and absence of a randomized comparator preclude conclusions regarding comparative or long-term safety.

Tumor biology was strongly associated with nodal response. The ypN0 rate was 20.5% in HR+/HER2− tumors, increasing to 40.5% in HR+/HER2+ disease, 56.5% in HR−/HER2+ disease, and 72.2% in TNBC. Radiologic nodal clearance followed a similar biological gradient. This pattern is consistent with the established relationship between treatment-responsive subtypes, pathological complete response, and improved outcomes after neoadjuvant therapy [29]. All patients with HER2-positive disease received standard dual HER2 blockade. Pembrolizumab was not routinely used in the TNBC subgroup during the study period. Current immunotherapy-containing regimens may therefore achieve higher nodal response rates and expand the proportion of patients potentially eligible for response-adapted axillary staging. The absence of routine pembrolizumab does not invalidate the observed association between radiologic response and nodal pathology, but it may limit the applicability of the absolute response rates to contemporary TNBC practice.

Reliable localization and retrieval of the initially metastatic index node are prerequisites for this approach. Carbon tattooing is a practical and relatively accessible technique that does not require radioactive seeds or specialized intraoperative detection equipment. The MARI, TATTOO, and other axillary marking studies have demonstrated the feasibility of retrieving a marked node after neoadjuvant therapy [30,31,32,33]. In our cohort, the clipped node was identified and localized under ultrasound guidance in 55 of the 61 radN0 cases. When the clip could not be visualized confidently on ultrasound, CT-guided carbon tattooing provided a rescue localization method in the remaining six cases, allowing successful retrieval of the target node. Such an alternative may prevent abandonment of a planned TAD procedure solely because the clip is no longer readily visible after treatment. Prospective TAXIS data and reports of clip displacement or marker loss further emphasize the need for a robust localization strategy [34,35,36]. Post-treatment ultrasound therefore contributes not only to assessment of nodal morphology but also to procedural planning and targeted-node localization [37].

Several limitations should be acknowledged. The retrospective, single-center design introduces selection bias and restricts generalizability. Selection for limited SLNB/TAD was response-adapted and therefore differed systematically from selection for ALND. Moreover, pathological verification was not uniform across surgical groups. In patients managed with limited SLNB/TAD without ALND, pathological assessment was restricted to the retrieved sentinel and targeted nodes; the remaining non-retrieved axillary lymph nodes were not histologically examined. In contrast, patients undergoing ALND had substantially more extensive pathological assessment of the axilla. This differential verification limits direct comparison between surgical groups and prevents the absence of residual disease in the retrieved SLNB/TAD nodes from being interpreted as definitive evidence of complete sterilization of the entire axilla.

Although institutional practice was to retrieve at least three axillary lymph nodes during SLNB/TAD whenever technically feasible, one patient (1.9%) in the limited-surgery cohort had only one node retrieved. This isolated case should also be considered when interpreting the pathological findings of the limited-surgery group. Although examinations were reviewed in consensus by three breast radiologists with more than ten years of experience, formal interobserver reproducibility was not assessed. Detailed radiotherapy data and patient-reported outcomes, including lymphedema, shoulder function, pain, and quality of life, were not evaluated comparatively. Finally, the number of regional events was small and follow-up remains insufficient to establish long-term oncologic safety. Prospective multicenter studies should combine standardized radiologic response criteria with reliable target-node localization, clearly defined surgical and radiotherapy pathways, patient-reported morbidity outcomes, and long-term regional control.

Taken together, these findings position radN0 as a component of multidisciplinary decision-making rather than an independent indication for omitting ALND in patients with cN2 breast cancer. A favorable radiologic response may support proceeding with limited surgical staging using SLNB and targeted retrieval of the previously involved node, provided that biological, clinical, and intraoperative findings are also considered. Thus, initial cN2 status should not automatically exclude well-selected responders from a less invasive axillary approach.

## 5. Conclusions

Clinically meaningful nodal downstaging can occur after neoadjuvant treatment in patients presenting with cN2 breast cancer. Radiologic nodal clearance was strongly associated with pathologically node-negative findings and may help identify patients suitable for SLNB/TAD-based staging within a multidisciplinary response-adapted pathway. However, radN0 should not replace surgical staging or independently determine the extent of axillary surgery. These findings support integrating post-treatment imaging with tumor biology, sentinel mapping, confirmed retrieval of the previously involved target node, and intraoperative assessment. Prospective studies with standardized treatment pathways and longer oncologic follow-up are required before routine omission of ALND can be recommended in this cN2 population.

## Figures and Tables

**Table 1:** Clinicopathologic and treatment-response characteristics according to post-treatment radiologic nodal status.

Characteristic	Overall (*n* = 166)	radN0 (*n* = 61)	radN+ (*n* = 105)	*p* Value
Age, years, mean ± SD	49.0 ± 11.7	48.4 ± 12.6	49.3 ± 11.2	0.626
**Histologic subtype**				0.119
NST	149 (89.8)	57 (93.4)	92 (87.6)	
ILC	10 (6.0)	4 (6.6)	6 (5.7)	
Rare variants	7 (4.2)	0	7 (6.7)	
**Molecular subtype**	** **	** **	** **	**<0.001**
HR+/HER2−	88 (53.0)	23 (37.7)	65 (61.9)	
HR+/HER2+	37 (22.3)	12 (19.7)	25 (23.8)	
HR−/HER2+	23 (13.9)	13 (21.3)	10 (9.5)	
TNBC	18 (10.8)	13 (21.3)	5 (4.8)	
**Primary tumor radiologic response**	** **	** **	** **	**<0.001**
Complete response	36 (21.7)	36 (59.0)	0	
Partial response	112 (67.5)	23 (37.7)	89 (84.8)	
Stable disease	15 (9.0)	2 (3.3)	13 (12.4)	
Progressive disease	3 (1.8)	0	3 (2.9)	
**Residual cancer burden**	** **	** **	** **	**<0.001**
RCB-0	35 (21.1)	35 (57.4)	0	
RCB-I	16 (9.6)	8 (13.1)	8 (7.6)	
RCB-II	60 (36.1)	17 (27.9)	43 (41.0)	
RCB-III	55 (33.1)	1 (1.6)	54 (51.4)	
**Breast surgery**	** **	** **	** **	**0.379**
Breast-conserving surgery	103 (62.0)	41 (67.2)	62 (59.0)	
Mastectomy	63 (38.0)	20 (32.8)	43 (41.0)	

Note: Data are presented as *n* (%) unless otherwise indicated. *p* values compare the radN0 and radN+ groups and were calculated using the independent-samples *t*-test, chi-square test, or Fisher’s exact test, as appropriate. Abbreviations: HER2, human epidermal growth factor receptor 2; HR, hormone receptor; ILC, invasive lobular carcinoma; NST, invasive carcinoma of no special type; RCB, residual cancer burden; SD, standard deviation; TNBC, triple-negative breast cancer.

**Table 2:** Final axillary surgical management and pathological nodal outcomes according to post-treatment radiologic nodal status.

Outcome	Overall (*n* = 166)	radN0 (*n* = 61)	radN+ (*n* = 105)
**Final axillary procedure**			
Limited SLNB/TAD without ALND	53 (31.9)	47 (77.0)	6 (5.7)
Limited surgery followed by completion ALND	14 (8.4)	6 (9.8)	8 (7.6)
Primary ALND	99 (59.6)	8 (13.1)	91 (86.7)
**Final pathological nodal stage**			
ypN0	59 (35.5)	53 (86.9)	6 (5.7)
ypN0(i+)	4 (2.4)	3 (4.9)	1 (1.0)
ypN1	61 (36.7)	5 (8.2)	56 (53.3)
ypN2	27 (16.3)	0	27 (25.7)
ypN3	15 (9.0)	0	15 (14.3)

Note: Data are presented as *n* (%). Percentages in the radN0 and radN+ columns were calculated within each radiologic nodal group. Axillary pathological complete response (pCR) was defined as ypN0(i−); cases with isolated tumor cells [ypN0(i+)] were classified separately. Abbreviations: ALND, axillary lymph node dissection; pCR, pathological complete response; radN0, radiologic node-negative status; radN+, persistent radiologically suspicious nodal disease; SLNB, sentinel lymph node biopsy; TAD, targeted axillary dissection.

**Table 3:** Pathological nodal outcomes and number of excised nodes according to the final axillary surgical procedure.

Outcome	Limited SLNB/TAD (*n* = 53)	Completion ALND (*n* = 14)	Primary ALND (*n* = 99)
**Pathological nodal stage**			
ypN0	43 (81.1)	2 (14.3)	14 (14.1)
ypN0(i+)	3 (5.7)	0	1 (1.0)
ypN1	7 (13.2)	12 (85.7)	42 (42.4)
ypN2	0	0	27 (27.3)
ypN3	0	0	15 (15.2)
**Excised nodes, mean ± SD**	4.6 ± 1.9	12.1 ± 4.0	16.9 ± 5.3
**Excised nodes, median (range)**	4 (1–9)	10.5 (8–20)	17 (6–28)

Note: Data are presented as *n* (%) unless otherwise indicated. Axillary pathological complete response was defined as ypN0(i−); cases with isolated tumor cells [ypN0(i+)] were classified separately. Differences among procedural groups reflect multidisciplinary and intraoperative selection and should not be interpreted as comparative effectiveness of the surgical procedures. Abbreviations: ALND, axillary lymph node dissection; SD, standard deviation; SLNB, sentinel lymph node biopsy; TAD, targeted axillary dissection.

**Table 4:** Post-treatment radiologic nodal clearance and pathological nodal complete response according to molecular subtype.

Molecular Subtype	Total, *n*	radN0, *n* (%)	ypN0, *n* (%)
HR+/HER2−	88	23 (26.1)	18 (20.5)
HR+/HER2+	37	12 (32.4)	15 (40.5)
HR−/HER2+	23	13 (56.5)	13 (56.5)
TNBC	18	13 (72.2)	13 (72.2)
*p* value		<0.001	<0.001

Note: Percentages were calculated within each molecular subtype. *p* values compare the four molecular subtypes. Abbreviations: HER2, human epidermal growth factor receptor 2; HR, hormone receptor; radN0, radiologic node-negative status; TNBC, triple-negative breast cancer; ypN0, axillary pathological complete response.

## Data Availability

The datasets generated and analyzed during the current study are not publicly available due to institutional privacy restrictions but are available from the corresponding author upon reasonable request.

## Supplementary Materials

The following supporting information can be downloaded at: https://www.mdpi.com/article/10.3390/cancers18172874/s1, Table S1. Radiologic–pathologic discordant cases. False-negative: radN0/ypN+; false-positive: radN+/ypN0. radN0, radiologic nodal clearance; radN+, persistent suspicious nodes after NAC; ypN, final pathologic nodal stage.

## Abbreviations

ALND (Axillary Lymph Node Dissection), SLNB (Sentinel Lymph Node Biopsy), TAD (Targeted Axillary Dissection), NAC (Neoadjuvant Chemotherapy), radN0 (Radiologic Node-Negative Status), radN+ (Radiologic Node-Positive Status), ypN0 (Pathologic Node-Negative Status after Neoadjuvant Therapy), ypN0(i+) (Pathologic Node-Negative Status with Isolated Tumor Cells after Neoadjuvant Therapy), ypN (Post-Neoadjuvant Pathologic Nodal Stage), HR (Hormone Receptor), HER2 (Human Epidermal Growth Factor Receptor 2), TNBC (Triple-Negative Breast Cancer), NST (Invasive Carcinoma of No Special Type), ILC (Invasive Lobular Carcinoma), RCB (Residual Cancer Burden), CT (Computed Tomography), FDG-PET/CT (Fluorodeoxyglucose Positron Emission Tomography/Computed Tomography), MRI (Magnetic Resonance Imaging), RECIST (Response Evaluation Criteria in Solid Tumors), ASCO (American Society of Clinical Oncology), CAP (College of American Pathologists), STROBE (Strengthening the Reporting of Observational Studies in Epidemiology), SD (Standard Deviation).
