# Supplementary material for: Radiologic Axillary Response After Neoadjuvant Chemotherapy in cN2 Breast Cancer: Decision Support for Selective Axillary De-Escalation
Source: Cancers (Basel). 2026 Sep 5;18(17):2874. doi: 10.3390/cancers18172874 (PMC13564531; doi:10.3390/cancers18172874)
Supplement: Supplementary file 1 [file cancers-18-02874-s001.zip › cancers-4527656-supplementary.pdf]

Supplementary Table S1. Radiologic–pathologic discordant cases**.** False-negative: radN0/ypN+; false-positive: radN+/ypN0. radN0, radiologic nodal clearance; radN+, persistent suspicious nodes after NAC; ypN, final pathologic nodal stage.

**Parameter**	**False-Negative (radN0 / ypN+)**	**False-Positive (radN+ / ypN0)**
Number of cases	5 (4.9%)	7 (4.2%)
Final ypN stage	All ypN1	All ypN0
Median positive node count	1	0
Molecular subtype distribution	4 Luminal (HER2−), 1 HER2+	4 HER2+, 2 Luminal (HER2−), 1 Triple-negative
Axillary recurrence	0	0
Clinical implication	Low-volume residual disease	Potential overtreatment risk

Note:For diagnostic-performance analyses, ypN0 and ypN0(i+) were categorized as pathologically negative, whereas ypN1–3 were categorized as pathologically positive. Strict nodal pCR was defined separately as ypN0(i−).
